# MicroRNA-214-3p targets the PLAGL2-MYH9 axis to suppress tumor proliferation and metastasis in human colorectal cancer

**DOI:** 10.18632/aging.103233

**Published:** 2020-05-15

**Authors:** Zili Zhou, Liang Wu, Zhengyi Liu, Xudan Zhang, Shengbo Han, Ning Zhao, Haijun Bao, Wenzheng Yuan, Jinhuang Chen, Jintong Ji, Xiaogang Shu

**Affiliations:** 1 Department of Gastrointestinal Surgery, Union Hospital, Tongji Medical College, Huazhong University of Science and Technology, Wuhan 430022, China; 2 Department of Breast Surgery, Henan Provincial People's Hospital, The People's Hospital of Zhengzhou University, The People's Hospital of Henan University, Zhengzhou 450003, China; 3 Department of Gastrointestinal Surgery II, Renmin Hospital of Wuhan University, Wuhan 430060, China; 4 Department of Emergency Surgery, Union Hospital, Tongji Medical College, Huazhong University of Science and Technology, Wuhan 430022, China; 5 Department of Gastrointestinal Surgery, The Central Hospital of Wuhan, Tongji Medical College, Huazhong University of Science and Technology, Wuhan 430022, China

**Keywords:** miR-214-3p, PLAGL2, colorectal cancer, proliferation, metastasis

## Abstract

Evidence has shown that microRNAs (miRNAs) participate in the progression of CRC. Previous studies have indicated that miR-214-3p is abnormally expressed in various malignant tumors. However, the biological function it plays in CRC and the potential mechanism are unclear. Here, we demonstrated that miR-214-3p was obviously downregulated in CRC. Moreover, we found a strong correlation between the miR-214-3p level and tumor size and lymphatic metastasis. Furthermore, when miR-214-3p was decreased by an Lv-miR-214-3p inhibitor, the proliferation and migration of SW480 and HCT116 cells were significantly increased. As expected, the ability of proliferation and migration was significantly suppressed when miR-214-3p was overexpressed in DLD1 cells. According to the dual-luciferase reporter results, PLAGL2 was found to be a direct downstream molecule of miR-214-3p. Chromatin immunoprecipitation (CHIP) confirmed that MYH9, a well-known cytoskeleton molecule in CRC, was a direct targeting gene of PLAGL2. Silencing PLAGL2 or MYH9 could reverse the effect of a miR-214-3p inhibitor on CRC cells. In summary, our studies proved that low expression of miR-214-3p and overexpression of downstream PLAGL2 in CRC indicated a poor prognosis. MiR-214-3p suppressed the malignant behaviors of colorectal cancer by regulating the PLAGL2/MYH9 axis. MiR-214-3p might be a novel therapeutic target or prognostic marker for CRC.

## INTRODUCTION

Colorectal cancer (CRC), the third most common cancer in the world, is a great challenge facing mankind [1]. The past decade has witnessed a significant increase in the incidence and mortality of CRC and a trend of occurrence in younger patients [2]. Although improvements in clinical diagnosis and comprehensive therapy have partly prolonged survival, the incidence and mortality of colorectal cancer are still high. The etiology and pathogenesis of colorectal cancer are not fully understood, but environmental, ethnic, economic and genetic factors play an important role in the progression of CRC. The high mortality and recurrence rates and the poor prognosis of colorectal cancer seriously threaten human safety and quality of life.

MicroRNAs (miRNAs), a class of endogenous non-coding small RNAs of approximately 22 nucleotides, suppress the transcription of target genes by directly binding to the 3’ non-coding region (UTR) of downstream genes [3, 4]. Increasing evidence has shown that microRNAs play a critical role in the pathophysiological processes of different cancers. MiR-328-3p promotes stemness and migration in ovarian cancer by targeting DDB2 [5]. MiR-204-5p inhibits metastasis in breast cancer by regulating PI3K/Akt signaling [6].

MiR-214-3p, located on chromosome 1, is abnormally expressed in various cancers and functions as an oncogene or tumor suppressor gene in different tumors [7]. Previous studies have revealed that miR-214-3p serves as an oncogene in pancreatic carcinoma and stomach adenocarcinoma. MiR-214-3p promotes the malignant behaviors of stomach adenocarcinoma through the Warburg effect and impairs the effect of chemotherapy in pancreatic cancer [8, 9]. Conversely, miR-214-3p is downregulated in breast carcinoma, colorectal cancer and liver hepatocellular carcinoma [10, 11]. MiR-214-3p inhibits autophagy by targeting UCP2 in breast carcinoma and inhibits the proliferation of hepatocellular carcinoma through the downregulation of MELK. Moreover, several studies have suggested that miR-214-3p is abnormally expressed in colorectal cancer and plays a significant role in the progression of CRC. MiR-214-3p suppresses the proliferation and migration of colon cancer by suppressing BCL9L, HSP27 and wnt signaling [12, 13]. However, the potential mechanism of miR-214-3p in cancer proliferation and migration has not been fully explored.

With the help of miRNA-related databases, polymorphic adenoma-like protein 2 (PLAGL2) has been demonstrated to be downstream of miR-214-3p; thus, miR-214-3p can directly bind to the 3’UTR of PLAGL2. A member of the PLAG gene family, PLAGL2, a zinc finger protein, is unregulated and plays an oncogenic role in several malignant tumors, such as breast cancer and bladder cancer [14, 15]. In colorectal cancer, the expression of PLAGL2 is obviously increased and acts as a tumor promoting factor [16–18]. Although some studies have revealed that PLAGL2 promotes migration and proliferation through wnt signaling [16], the underlying mechanisms of PLAGL2 in the regulation of CRC need further exploration.

Here, our research demonstrated that miR-214-3p inhibits the growth and metastasis of colorectal cancer by targeting PLAGL2. MYH9, a well-known cytoskeleton molecule, is closely related to the proliferation and migration of colorectal cancer and participates in the EMT process [19]. Considering that PLAGL2 also plays a role in the regulation of the actin cytoskeletal architecture and EMT process [18, 20], our studies indicated that the expression of MYH9 is correlated with PLAGL2. MYH9 is a direct downstream target of PLAGL2. Furthermore, we found that the miR-214-3p/PLAGL2/MYH9 axis has a significant effect on proliferation and metastasis in CRC. Taken together, our studies provide an explanation for the metastasis of CRC and might result in novel therapeutic targets or prognostic markers for CRC.

## RESULTS

### MiR-214-3p is downregulated in CRC tissues and associated with CRC metastasis and proliferation

**Figure 1:**
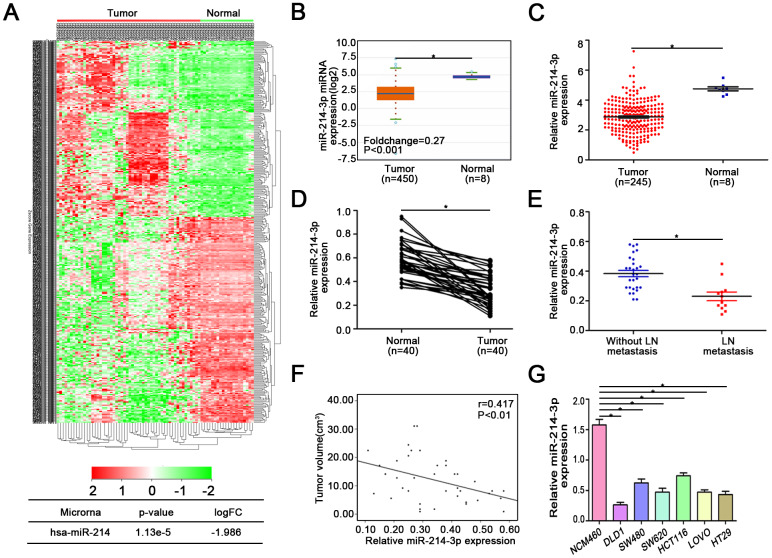
**MiR-214-3p is downregulated in CRC tissues and associated with CRC metastasis and proliferation.** (**A**) GEO, Starbase 3.0 (**B**), and (**C**) TCGA databases indicated that miR-214-3p is obviously downregulated in CRC. (**D**) The expression of miR-214-3p was lower in CRC tissues than in adjacent normal tissues. (**E**–**F**) The expression level of miR-214-3p was negatively correlated with lymph node metastasis and tumor size. (**G**) MiR-214-3p was significantly lower in CRC cells than in NCM460 cells. The data are represented as the means±S.D. from at least three independent experiments. *p<0.05.

According to the data from ONCOMIR [21] (Supplementary Figure 1), there are 10 cancer types, including colon cancer, in which tumorigenesis is significantly associated with the expression of miR-214-3p. We also found that miR-214-3p was significantly downregulated in colon cancer. Similarly, data from the TCGA, Starbase v3.0 [22] and GEO databases (GSE30454) also support this result (Figure 1A–1C). Then, qRT-PCR was used to analyze the expression of miR-214-3p in 40 paired CRC tissues and their controls (Figure 1D). Confirming the above database data, we found that the expression of miR-214-3p was lower in CRC tissues than in adjacent normal tissues. Remarkably, our results showed that the expression level of miR-214-3p was negatively correlated with lymph node metastasis and tumor size (Figure 1E–1F, Table 1). Thus, CRC tissues associated with lymph node metastasis and large tumor size contained lower expression of miR-214-3p. In addition, we compared the expression level of miR-214-3p between different cell lines (Figure 1G). The results also showed a significantly lower expression of miR-214-3p in CRC cells (DLD1, SW480, SW620, HCT116 and HT29) than in normal colonic epithelial cells (NCM460).

**Table 1:** Correlation of miR-214-3p and clinicopathological feature.

**Clinicopathological factors**	**n**	**MiR-214-3p expression**		**P value**
		**high**	**low**	
**Gender**				
Male	19	8	11	0.370
Female	21	6	15	
**Age, years**				
>60	21	7	14	0.816
≤60	19	7	12	
**Pathologic T stage**				
T1 + T2	16	8	8	0.104
T3 + T4	24	6	18	
**Pathologic N stage**				
N0	11	9	7	0.021
N1 + N2	29	5	19	
**Pathologic M stage**				
M0	23	11	12	0.048
M1	17	3	14	
**Tumor size**				
≤3 cm	23	12	11	0.008
>3cm	17	2	15	

### MiR-214-3p suppresses CRC cell proliferation and metastasis

**Figure 2:**
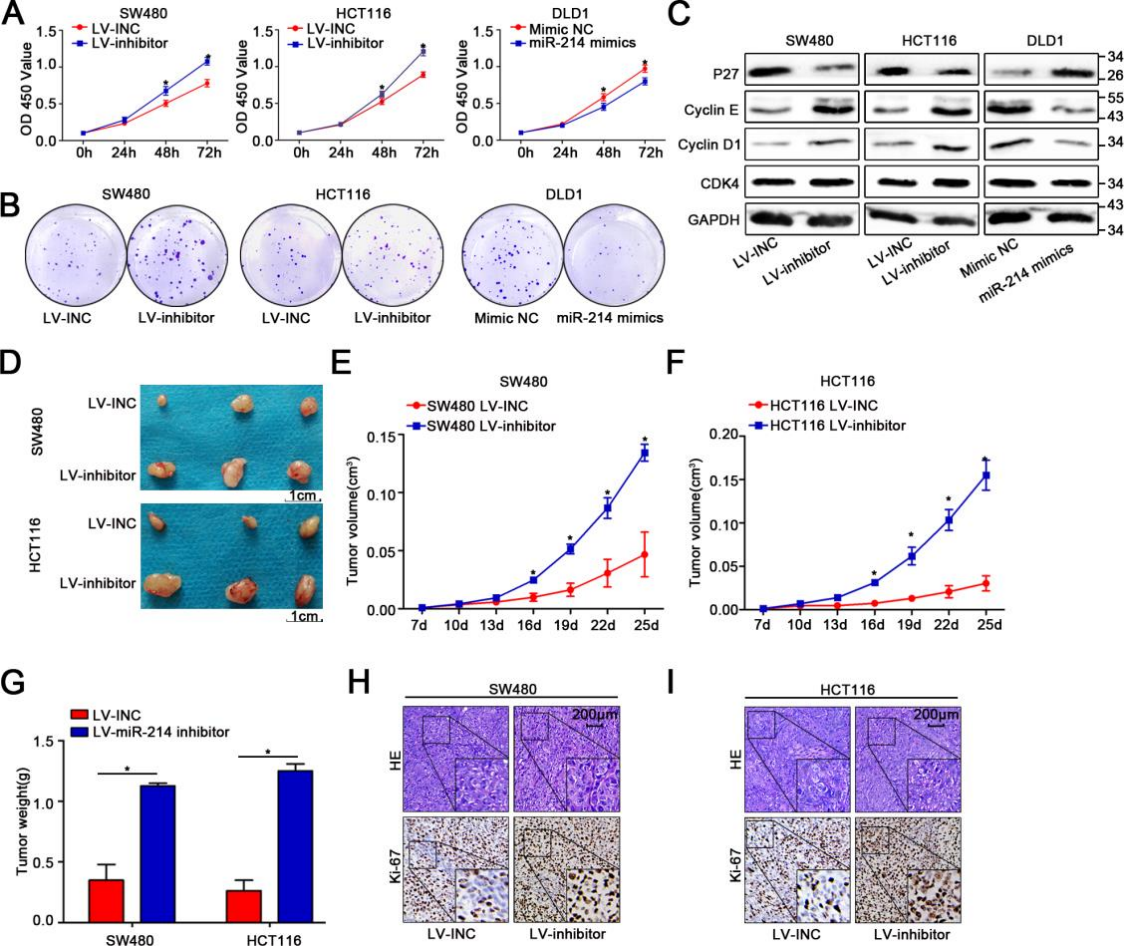
**MiR-214-3p suppresses CRC cell proliferation *in vitro* and *in vivo*.** (**A**–**B**) CCK8 and colony-formation assays indicated that miR-214-3p inhibited the proliferation of CRC cells. (**C**) Western blot assays showed that miR-214-3p decreased the expression of cyclin D1, cyclin E and CDK4 and increased the expression of P27. (**D**–**G**) Tumors grew faster in the LV-miR-214-3p inhibitor group than in the LV-INC group. The tumor weights in the LV-miR-214-3p inhibitor group were higher than those in the LV-INC group. (**H**–**I**) IHC results indicated that the Ki-67 (a proliferation marker) index was increased remarkably when miR-214-3p was knocked down. The data are represented as the means±S.D. from at least three independent experiments. *p<0.05.

The three colon cancer cell lines SW480, HCT116 and DLD1 were utilized to conduct the following experiments due to the previous results showing that miR-214-3p expression was lower in SW480 and HCT116 cells than in other colon cancer cell lines and higher in DLD1 cells than in other colon cancer cell lines. Therefore, a lentivirus-based system (LV-miR-214-3p inhibitor and LV-INC) was used to establish stable miR-214-3p knockdown SW480 and HCT116 cell lines. The efficiency of transfection was detected by qRT-PCR (Supplementary Figure 2A). CCK8, EDU and colony formation assays were applied (Figure 2A–2B, Supplementary Figure 2B–2E), and the results indicated that the proliferation ability was enhanced in SW480 and HCT116 cells when the expression of miR-214-3p was suppressed. The WB results also showed that the expression levels of the proliferation-related genes cyclin D1, cyclin E and CDK4 were increased, while the level of P27 was decreased when miR-214-3p was suppressed (Figure 2C, Supplementary Figure 2F). Additionally, the experiments above were repeated when miR-214-3p was overexpressed in DLD1 cells by transfection with the miR-214-3p mimic (Figure 2A–2C, Supplementary Figure 2A–2B, 2G); the results also contributed to the conclusion that miR-214-3p participated in suppressing proliferation in CRC cells. As expected, miR-214-3p knockdown significantly promoted the growth of subcutaneous xenograft tumors *in vivo* (Figure 2D–2G). The following IHC results indicated that the Ki-67 (a proliferation marker) index was increased remarkably when miR-214-3p was knocked down (Figure 2H–2I).

**Figure 3:**
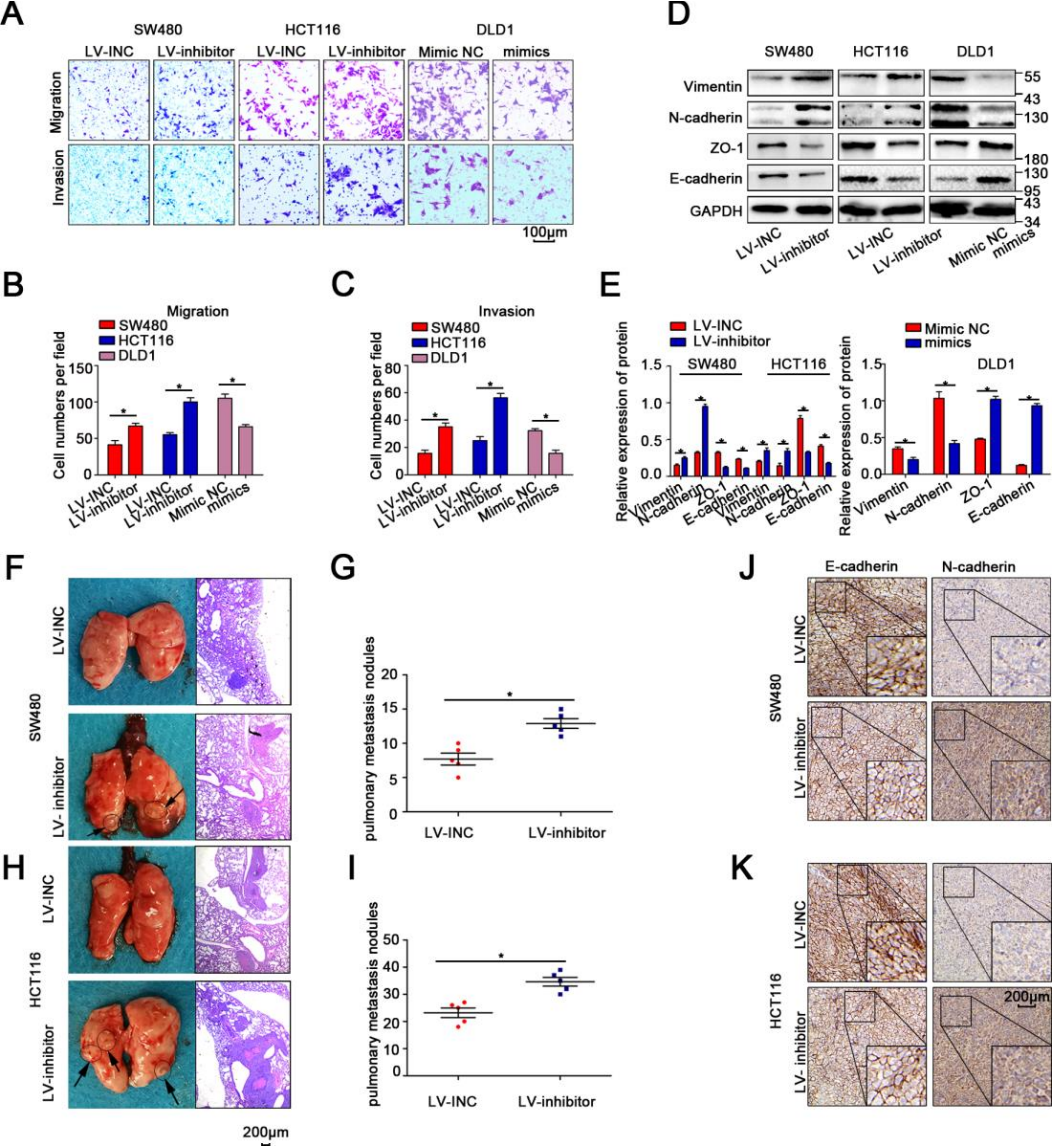
**MiR-214-3p suppresses CRC cell metastasis *in vitro* and *in vivo*.** (**A**–**C**) Transwell assays revealed that miR-214-3p suppressed CRC cell migration and invasion abilities. (**D**–**E**) Western blot analysis revealed that miR-214-3p decreased the expression of N-cadherin and vimentin and increased the expression of E-cadherin and Zo1. (**F**–**I**) More lung metastatic nodules were observed in the LV-miR-214-3p group than in the LV-INC group. (**J**–**K**) IHC analysis indicated that the expression of E-cadherin was lower and that the expression of N-cadherin was higher in xenograft subcutaneous tissues from the LV-miR-214-3p inhibitor group. The data are represented as the means±S.D. from at least three independent experiments. *p<0.05.

Transwell and wound-healing assays indicated that migration was enhanced when miR-214-3p expression was suppressed in SW480 and HCT116 cells (Figure 3A–3C, Supplementary Figure 3A–3B). Moreover, WB, qRT-PCR and IF demonstrated that the suppression of miR-214-3p could significantly increase the levels of E-cadherin and Zo1, as well as inhibit the expression of N-cadherin and vimentin (Figure 3D–3E, Supplementary Figure 3C–3D). Additionally, the results in DLD1 cells when miR-214-3p was overexpressed via miR-214-3p mimic also supported the conclusion that miR-214-3p overexpression significantly suppressed cell migration (Figure 3A–3E, Supplementary Figure 3C). To explore the effect of miR-214-3p on metastasis activity *in vivo*, SW480 and HCT116 cells transfected with the LV-miR-214-3p inhibitor or LV-INC were injected into the tail vein of immunodeficient mice (Figure 3F–3I). The results showed that more pulmonary metastasis nodules could be observed in mice injected with the LV-miR-214-3p inhibitor. Furthermore, we measured the expression of EMT-association proteins in the subcutaneous xenograft tumors by IHC analysis (Figure 3J–3K), and the results showed that the expression of epithelial markers (e.g., E-cadherin) was significantly decreased and that the expression of mesenchymal markers (e.g., N-cadherin) was significantly inhibited when miR-214-3p was knocked down. In summary, the data above support the conclusion that miR-214-3p suppressed CRC cell proliferation and migration *in vitro* and *in vivo*.

### MiR-214-3p directly targets PLAGL2 mRNA

**Figure 4:**
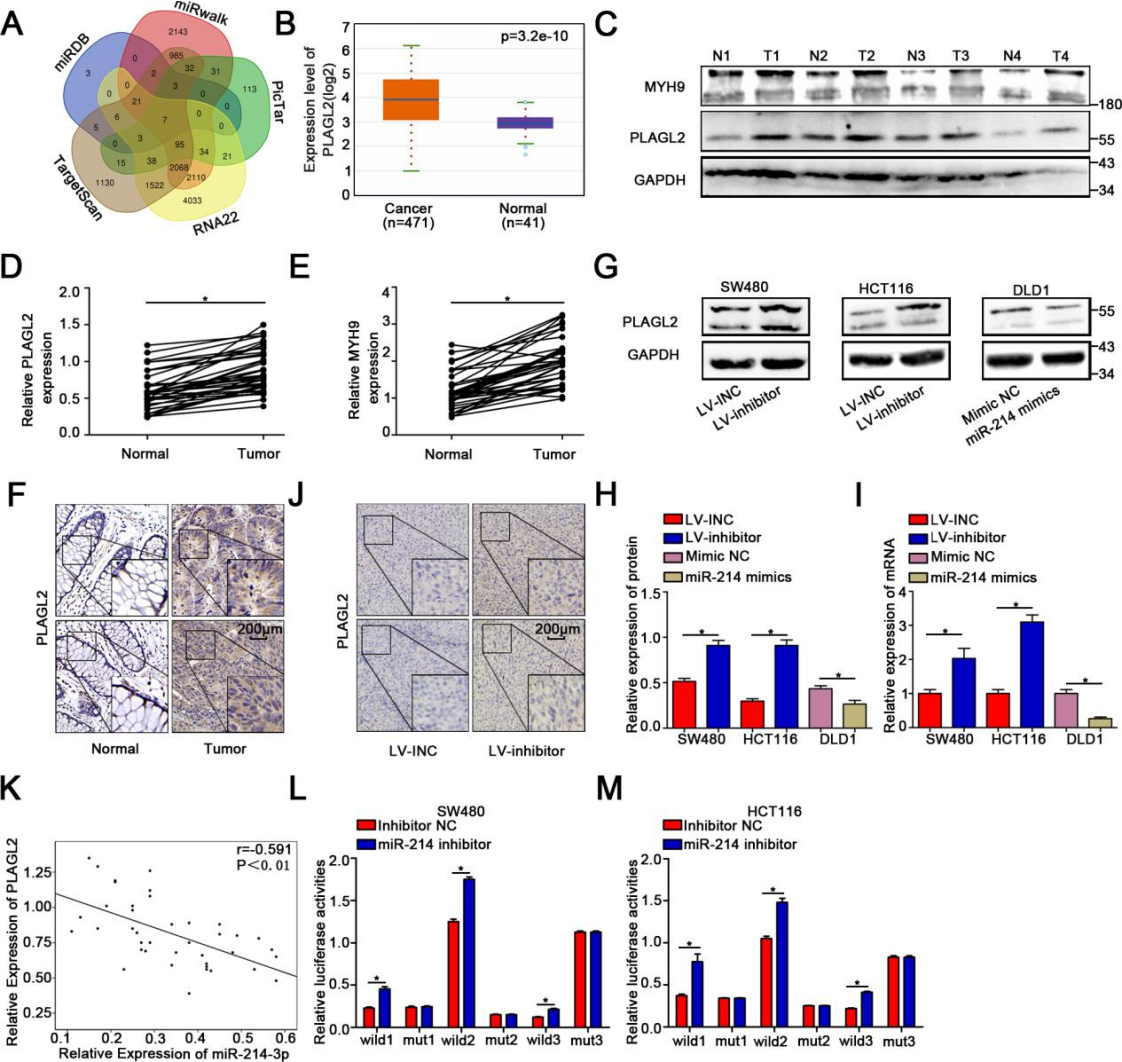
**MiR-214-3p directly targets PLAGL2.** (**A**) Based on the public bioinformatics algorithms, seven potential targets of miR-214-3p were selected. (**B**–**F**) The Starbase 3.0 database and our study indicated that the expression levels of PLAGL2 and MYH9 were higher in cancer tissues than in normal tissues. (**G**–**I**) The mRNA and protein levels of PLAGL2 were inhibited by miR-214-3p. (**J**) IHC analysis indicated that the expression of PLAGL2 could be inhibited by miR-214-3p in xenograft subcutaneous tissues. (**K**) qRT-PCR analysis demonstrated that PLAGL2 was negatively correlated with miR-214-3p expression in CRC tissues. (**L**–**M**) Dual luciferase reporter assays in CRC cells. The data are represented as the means±S.D. from at least three independent experiments. *p<0.05.

To further explore the downstream genes of miR-214-3p, five public bioinformatics algorithms, TargetScan [23], miRDB [24] (the top fifty predicted genes were chosen), miRwalk [25], miR22 [26] and Pictar [27], were utilized. We found seven candidate target genes of miR-214-3p (Figure 4A). MicroRNAs play an inhibitory role in the regulation of direct target genes. GEPIA (http://gepia.cancer-pku.cn/) was applied to compare the expression levels of the seven candidate genes in CRC tissues and adjacent normal tissues (Figure 4B, Supplementary Figure 4A–4F). The results showed that PLAGL2 expression was obviously high in CRC tissues, indicating that PLAGL2 is likely directly regulated by miR-214-3p. The expression level of PLAGL2 in the CRC tissues we collected was also measured (Figure 4C–4D, Figure 4F); as expected, the level of PLAGL2 was obviously higher in the cancer tissues than in the normal tissues. Next, we found that the mRNA and protein levels of PLAGL2 in SW480 and HCT116 cells were significantly increased when miR-214-3p was suppressed. The opposite results were observed when miR-214-3p was overexpressed in DLD1 cells (Figure 4G–4J, Supplementary Figure 4G–4H). Next, we analyzed the correlation between the expression levels of miR-214-3p and PLAGL2 (Figure 4K). The results indicated that the expression level of PLAGL2 was frequently higher in samples with low miR-214-3p expression. Finally, dual-luciferase reporter assays were conducted (Figure 4L–4M, Supplementary Figure 4I). The relative luciferase activity of the PLAGL2 WT pmirGlo-3’-UTR vector was markedly increased when miR-214-3p was decreased in SW480 and HCT116 cells, whereas miR-214-3p was incapable of altering the luciferase activity of the PLAGL2-Mut pmirGlo-3’-UTR vector, indicating that miR-214-3p likely regulates the expression of PLAGL2 through direct binding to the 3’-UTR. Considering all the data above, we concluded that PLAGL2 is a direct downstream gene of miR-214-3p.

### PLAGL2 accelerates CRC cell growth and migration

**Figure 5:**
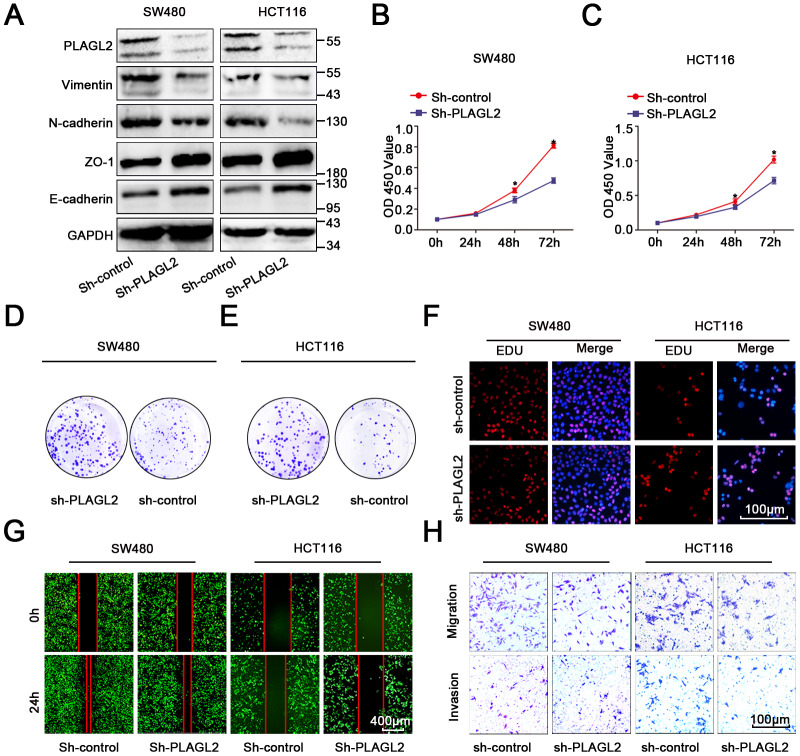
**PLAGL2 accelerates CRC cell growth and migration.** (**A**) WB indicated that downregulation of PLAGL2 could inhibit the expression of vimentin and N-cadherin and increase the expression of Zo-1 and E-cadherin. (**B**–**C**) CCK8 assays indicated that PLAGL2 promoted the proliferation of CRC cells. (**D**–**E**) Colony-formation assays indicated that PLAGL2 promoted the proliferation of CRC cells. (**F**) EDU assays indicated that PLAGL2 promoted the proliferation of CRC cells. (**G**–**H**) Transwell and wound-healing assays showed that PLAGL2 promoted the migration and invasion of CRC cells. The data are represented as the means±S.D. from at least three independent experiments. *p<0.05.

To further explore the function of PLAGL2 in colon cancer cells, we first transfected sh-PLAGL2 into SW480 and HCT116 cells to establish stable knockdown cell lines with the lentiviral vector. Then, we utilized WB and qRT-PCR assays to detect the efficiency of transfection (Figure 5A, Supplementary Figure 5A–5D). Next, we evaluated the EMT process when PLAGL2 was knocked down (Figure 5A, Supplementary Figure 5A–5D). We found that silencing PLAGL2 obviously inhibited the expression of epithelial markers and enhanced the expression of mesenchymal markers. Subsequently, we conducted CCK8, EDU and colony formation assays to evaluate the function of PLAGL2 in the proliferation of CRC cells (Figure 5B–5F, Supplementary Figure 5E–5F). Finally, transwell and wound-healing assays were conducted to investigate the function of PLAGL2 in the migration of CRC cells (Figure 5G–5H, Supplementary Figure 5G–5H). The results indicated that the knockdown of PLAGL2 could obviously decrease the growth ability and metastasis of CRC cells.

### Silencing PLAGL2 significantly reverses the malignant process caused by a miR-214-3p inhibitor in CRC

**Figure 6:**
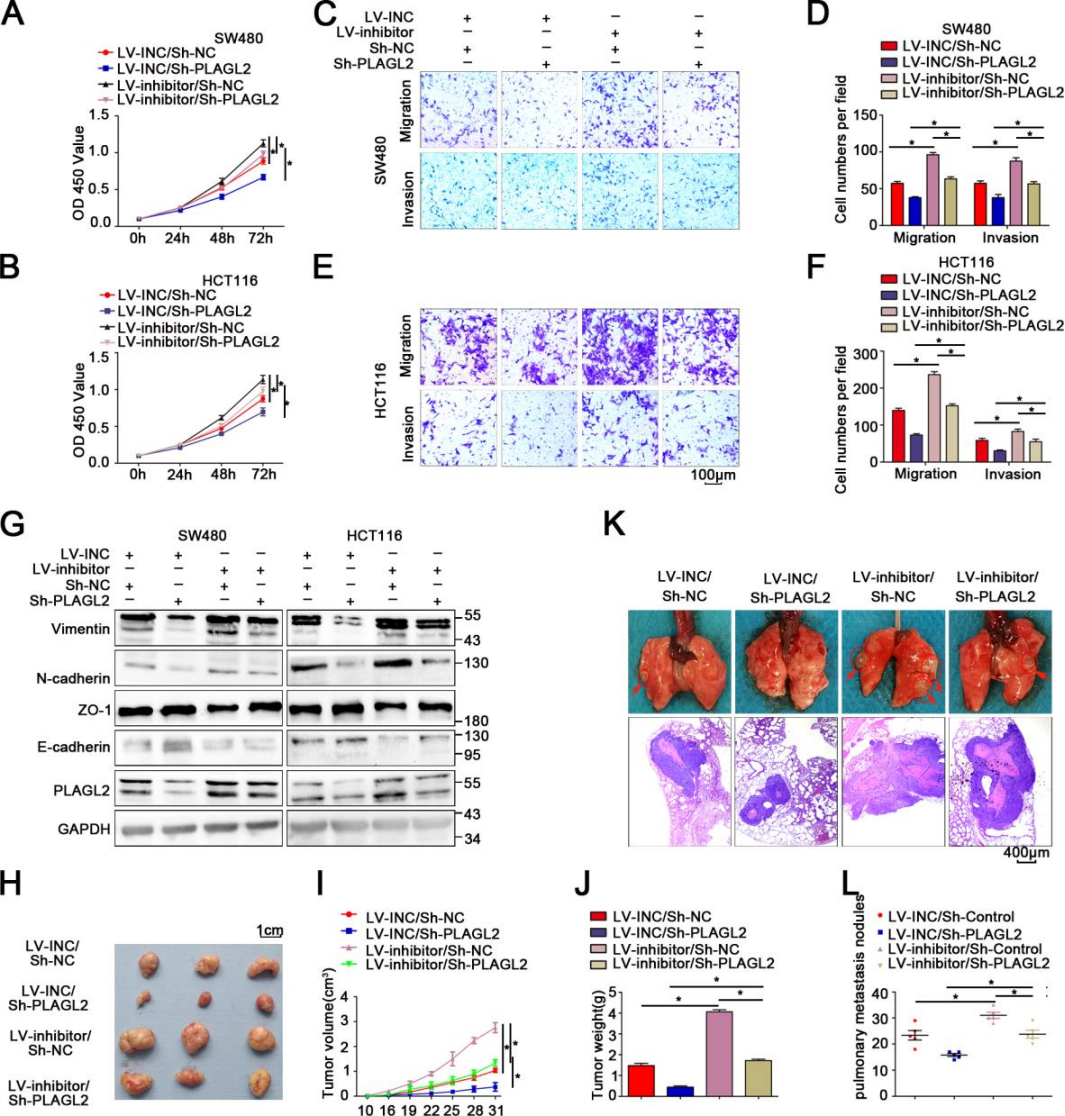
**Silencing PLAGL2 significantly reverses the malignant process caused by the miR-214-3p inhibitor in CRC.** (**A**–**F**) CCK8 and transwell assays indicated that PLAGL2 downregulation effectively reversed miR-214-3p inhibitor-induced proliferation (**A**–**B**) and the migration and invasion (**C**–**F**) abilities of CRC cells. (**G**) WB analysis revealed that the inhibitory effect of miR-214-3p on EMT was reversed by Sh-PLAGL2 transfection. (**H**–**J**) Silencing PLAGL2 reversed the effect of the miR-214-3p inhibitor on tumor growth *in vivo*. (**K**–**L**) Silencing PLAGL2 reversed the effect of the miR-214-3p inhibitor on tumor metastasis *in vivo*. The data are represented as the means±S.D. from at least three independent experiments. *p<0.05.

A rescue experiment was conducted to evaluate the regulatory role that PLAGL2 plays in miR-214-3p-mediated CRC progression. First, an Lv-miR-214-3p inhibitor and Sh-PLAGL2 were co-transfected to establish stable cell lines. Then, the following experiments were conducted. The results of the CCK8 and EDU assays indicated that the proliferation ability was enhanced in CRC cells when miR-214-3p expression alone was silenced when compared with that of cells when both miR-214-3p and PLAGL2 expression were inhibited (Figure 6A–6B, Supplementary Figure 6A–6B). Subsequently, transwell and wound-healing assays were conducted (Figure 6C–6F, Supplementary Figure 6C–6E), and the results indicated that silencing PLAGL2 could significantly reverse the promotion of migration caused by the miR inhibitor in CRC. What’s more, the EMT process caused by the miR inhibitor was also reversed when PLAGL2 was silenced (Figure 6G, Supplementary Figure 6F–6I). Furthermore, *in vivo* experiments indicated that knockdown of miR-214-3p along with sh-PLAGL2 weakened proliferation and migration compared to knockdown of miR-214-3p alone (Figure 6H–6L). Taken together, the data above show that silencing PLAGL2 could effectively reverse miR inhibitor-induced CRC progression *in vitro* and *in vivo*.

### MYH9 is a direct target gene regulated by PLAGL2

**Figure 7:**
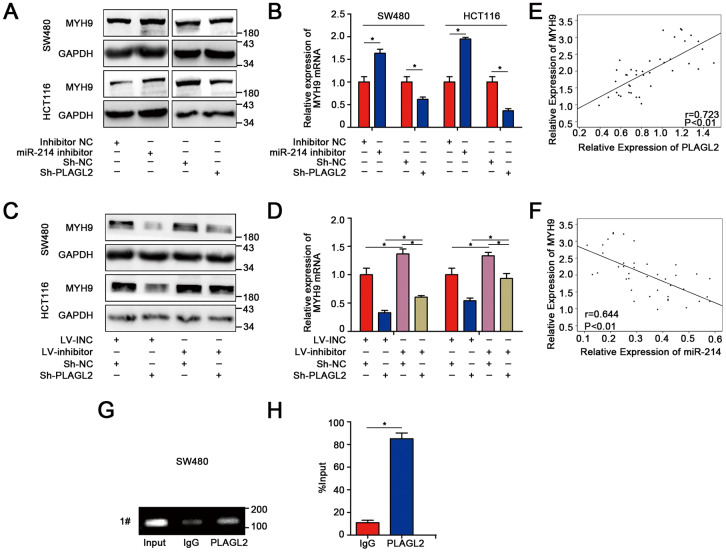
**MYH9 is a direct target gene regulated by PLAGL2.** (**A**–**B**) WB and qRT-PCR analyses indicated that the expression of MYH9 was regulated by miR-214-3p and PLAGL2. (**C**–**D**) The inhibitory effect of miR-214-3p on MYH9 could be reversed by Sh-PLAGL2. (**E**–**F**) qRT-PCR analysis demonstrated that MYH9 was correlated with the expression of miR-214-3p and PLAGL2 in CRC tissues. (**G**–**H**) ChIP assays with PLAGL2 antibody or IgG were performed to verify the binding between PLAGL2 and the MYH9 promoter in SW480 cells. The data are represented as the means±S.D. from at least three independent experiments. *p<0.05.

Previous studies have shown that PLAGL2 could regulate the actin cytoskeletal architecture. MYH9, a well-known cytoskeleton molecule, is closely related to the proliferation and metastasis of human CRC. Therefore, we attempted to explore the relationship between PLAGL2 and MYH9. Interestingly, we found that silencing PLAGL2 obviously decreased the expression of MYH9 at both the protein and mRNA levels. MYH9 could also be regulated by miR-214-3p (Figure 7A–7B). Furthermore, knockdown of PLAGL2 significantly reversed the suppression effect of the miR-214-3p inhibitor on the expression of MYH9 (Figure 7C–7D, Supplementary Figure 7B). Next, we investigated the expression level of MYH9 in CRC tissues and found that MYH9 was significantly higher in the cancer tissues than in the adjacent normal tissues (Figure 4C, 4E and Supplementary Figure 7A), which was consistent with previous results. The data also indicated that there was an obvious correlation between the expression levels of PLAGL2 and MYH9 (Figure 7E). Moreover, a significant correlation between the expression levels of miR-214-3p and MYH9 was also observed (Figure 7F). Finally, ChIP assays were performed in SW480 cells (Figure 7G–7H). Compared to the sample bound to IgG, the PLAGL2-bound complex showed a remarkable enrichment of the MYH9 promoter. Considering the data above, we propose that the expression of MYH9 was regulated by miR-214-3p and PLAGL2 and that MYH9 was a direct downstream gene of PLAGL2.

### MiR-214-3p targets the PLAGL2-MYH9 axis to suppress tumor proliferation and metastasis in human colorectal cancer

**Figure 8:**
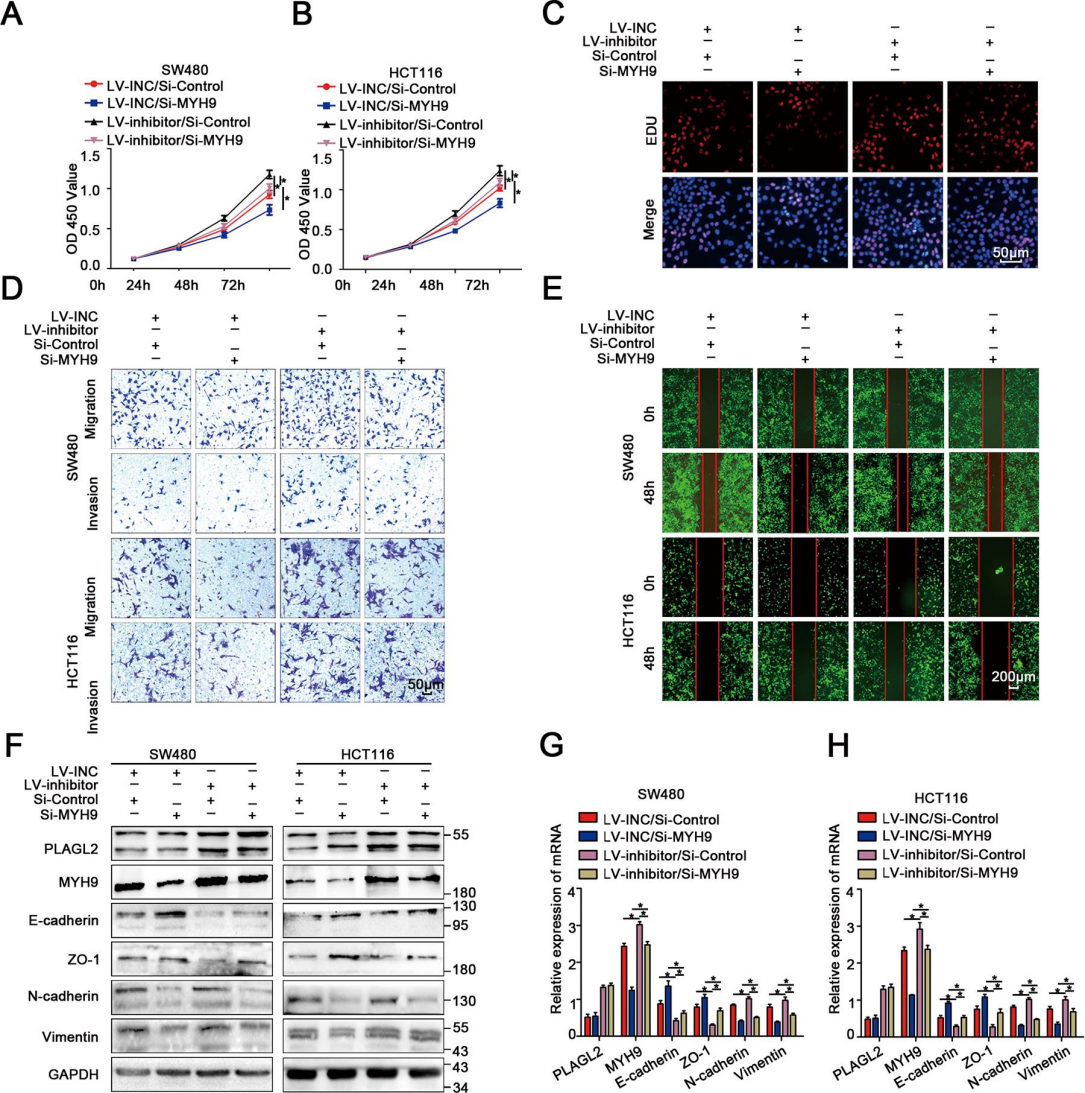
**MiR-214-3p targets the PLAGL2-MYH9 axis to suppress tumor proliferation and metastasis in human colorectal cancer.** (**A**–**B**) CCK8 assays indicated that PLAGL2 downregulation effectively reverses the miR-214-3p inhibitor-induced proliferation ability of CRC cells. (**C**) EDU assays indicated that PLAGL2 downregulation effectively reverses the miR-214-3p inhibitor-induced proliferation ability of CRC cells. (**D**–**E**) Transwell and wound-healing assays indicated that PLAGL2 downregulation effectively reversed miR-214-3p inhibitor-induced migration and invasion of CRC cells. (**F**–**H**) WB and qRT-PCR analyses revealed that the inhibitory effect of miR-214-3p on EMT was reversed by Si-MYH9 transfection. The data are represented as the means±S.D. from at least three independent experiments. *p<0.05.

To explore the biological function of MYH9 in miR-214-3p/PLAGL2-regulated colon cancer progression, si-MYH9 was co-transfected with the Lv-miR-214-3p inhibitor into SW480 and HCT116 cells. According to the results of CCK8 and EDU assays (Figure 8A–8C, Supplementary Figure 8A), MYH9 silencing could reverse the effects of miR-214-3p inhibition on CRC cell proliferation. We next utilized transwell and wound-healing assays to analyze the metastasis ability when si-MYH9 and Lv-miR-214-3p inhibitors were co-transfected (Figure 8D–8E, Supplementary Figure 8B–8E). The results showed that the CRC cells co-transfected with Lv-miR-214-3p inhibitor+si-MYH9 showed weaker migration ability than cells co-transfected with Lv-miR-214-3p inhibitor+si-NC. What’s more, the EMT process caused by the miR inhibitor was also reversed when MYH9 was silenced (Figure 8F–8H).

## DISCUSSION

Increasing evidence has revealed that miRNAs play critical roles in the occurrence and development of colon cancer [5, 6]. Until now, the underlying mechanism of miRNA in the proliferation and migration of colon cancer was unclear. Previous studies have indicated that miR-214-3p acts as an oncogene or tumor suppressor in various cancers [12, 13]. However, the biological function of miR-214-3p in CRC remains controversial. In this study, miR-214-3p was downregulated in CRC, and low expression of miR-214-3p was significantly associated with tumor size and lymphatic metastasis in CRC. According to the expression level of miR-214-3p in CRC cells. We suppressed the expression of miR-214-3p in SW480 and HCT116 cells, the results indicated that the ability of proliferation and migration was enhanced. Then, we overexpressed the expression of miR-214-3p in DLD1 cells, we found that the ability of proliferation and migration was significantly suppressed. In addition, dual-luciferase reporter assays indicated that miR-214-3p directly binding to the 3’UTR of PLAGL2. Then, MYH9 was demonstrated to be a direct target downstream of PLAGL2. Taken together, our studies indicate that miR-214-3p targeted the PLAGL2-MYH9 axis to suppress tumor proliferation and metastasis in human colorectal cancer.

Several studies have illustrated that miR-214-3p acts as a tumor suppressor in colon cancer. MiR-214-3p suppresses the proliferation and migration of colon cancer by suppressing BCL9L, HSP27 and wnt signaling [12, 13]. MiR-214-3p also participates in the tumorigenesis and stemness of colon cancer through the mTOR/β-catenin pathway [28]. Undoubtedly, miR-214-3p plays a critical role in the progression of CRC; however, the potential mechanism needs to be further explored. Here, we found that miR-214-3p was significantly decreased in colon cancer, and silencing miR-214-3p inhibited the proliferation and migration of human colon cancer cells *in vivo* and *in vitro*. Our findings are supported by other CRC experiments. However, the underlying mechanism through which miR-214-3p suppresses the progression of colon cancer remains unclear. In our current study, we investigated the role of miR-214-3p in the EMT process in colon cancer. Our results showed that downregulation of miR-214-3p decreased the expression of E-cadherin and Zo1 and increased the expression of vimentin and N-cadherin in CRC cells, indicating that miR-214-3p may promote the progression of CRC by regulating the EMT process. The mechanism by which the target gene miR-214-3p exerts its effect on EMT will be clarified in future studies. Biological analysis using five miRNA prediction databases was used to predict the potential target gene of miR-214-3p. Given that miR-214-3p was downregulated in CRC, PLAGL2 was selected as a candidate miR-214-3p target gene; we subsequently performed a dual-luciferase reporter gene assay, qRT-PCR, and Western blotting to verify this hypothesis.

PLAGL2, a well-known transcription factor, has been proposed to participate in the physiological regulation of different types of cancers [14, 15]. Previous studies have indicated that the expression of PLAGL2 was significantly higher in CRC tissues than in adjacent normal tissues and correlated with the depth of tumor invasion. Furthermore, PLAGL2 has been demonstrated to act as an oncogene in CRC by activating the Wnt6 and IGF2/β-catenin signaling pathways [16, 17]. Another study illustrated that PLAGL2 could regulate the actin cytoskeletal architecture and EMT process [18, 20]. In this study, we demonstrated that the expression of PLAGL2 was significantly correlated with that of miR-214-3p. Furthermore, we explored the role of PLAGL2 in the progression of CRC. Consistent with previous studies, our results indicated that silencing PLAGL2 could remarkably suppress the growth and migration of CRC cells. MYH9, a well-known cytoskeleton molecule, is closely related to the proliferation and metastasis of human colorectal cancer [19]. Given that PLAGL2 also participates in the regulation of the actin cytoskeletal architecture, we unsurprisingly demonstrated that MYH9 was directly transcriptionally regulated by PLAGL2. Our results add new evidence for the oncogenic function of PLAGL2 in the progression of CRC.

Our study provided robust evidence that miR-214-3p acts as a tumor-suppressor gene to inhibit CRC cell proliferation and migration by regulating the PLAGL2/MYH axis. However, increased expression of miR-214-3p may also increase the risk of other cancers, such as pancreatic carcinoma and stomach adenocarcinoma. So, what we next to do is to find out how to target miR-214-3p into colon cancer tissue specifically. A possible solution may be nano-drug carriers which can transporting drugs into tumor tissue specifically without any impact on normal tissues. We do believe that miR-214-3p can serve as a novel prognostic and diagnostic biomarker for CRC. This new signaling axis is a promising therapeutic strategy for CRC treatment.

## MATERIALS AND METHODS

### Patient tissues

CRC tissues and adjacent normal tissues from forty patients with no chemoradiotherapy before surgery were obtained at Wuhan Union Hospital between 2016 and 2018. Our research was approved by the Human Research Ethics Committee of Huazhong University of Science and Technology.

### Cell culture and reagents

SW480, HCT116 and DLD1 cells were cultured in DMEM (HyClone, Logan, UT, USA) mixed with 10% fetal bovine serum (FBS; Sciencell, Carlsbad, CA, USA) and maintained in a 5% CO2 humidified atmosphere at 37°C.

### Statistical analysis

The expression of miR-214-3p and PLAGL2 in 40 paired CRC tissues was analyzed by paired t test. Correlations among the expression levels of miR-214-3p, PLAGL2 and MYH9 in the matched colon cancer tissues were evaluated using Pearson’s r. A P value of ≤0.05 was considered statistically significant. The data analyses were carried out with SPSS and GraphPad Prism. The results are shown as the mean±standard deviation (SD). All tests were two-sided, and all experiments were repeated at least 3 times.

All supplementary methods are available in supplementary material.

## Supplementary Material

Supplementary Methods

Supplementary Figures

Supplementary Tables
