# Supplementary material for: MicroRNA-214-3p targets the PLAGL2-MYH9 axis to suppress tumor proliferation and metastasis in human colorectal cancer
Source: Aging (Albany NY). 2020 May 15;12(10):9633–57. doi: 10.18632/aging.103233 (PMC7288958; doi:10.18632/aging.103233)
Supplement: Supplementary Methods [file aging-12-103233-s001..pdf]

SUPPLEMENTARY METHODS

Oligonucleotides, siRNA, and lentiviral transfection

MiR-214-3p mimics, miR-214-3p inhibitors, and their corresponding control oligonucleotides were synthesized by RiboBio (Guangzhou, China). Transfection was carried out with a final concentration of 50 nM miR-214-3p mimics and 100 nM miR-214-3p inhibitors using the Lipofectamine 3000 reagent (Invitrogen, CA, USA) following the manufacturer's protocol. Lentiviral vector with miR-214-3p inhibitors and PLAGL2 shRNA was purchased from Genechem (Shanghai, China). which carried a green fluorescent protein sequence and puromycin sequence. SW480 and HCT116 were transfected with lentiviral vector with miR-214-3p inhibitors or PLAGL2 shRNA to establish stable cell lines with downexpression of miR-214-3p or PLAGL2. In order to select the stable cell lines, lentivirus-transfected cells were cultured in medium with 1 µg/ml puromycin for 14 days. MYH9 siRNA was obtained from RiboBio (Guangzhou, China). SW480 and HCT116 were transfected with MYH9 siRNA using Lipofectamine 3000 reagent (Invitrogen, CA, USA). The miRNA mimics, inhibitor, siRNAs were showed in Supplementary Table 1.

RNA isolation, quantitative real-time PCR (qPCR) and Western blot

Total RNA from tissue samples and cultured cells was extracted using TRIzol reagent (Invitrogen). Quantitative real-time PCR (qPCR) assays were carried out to detect mRNA expression using the PrimeScript RT Reagent Kit (TaKaRa) and SYBR Premix Ex Taq (TaKaRa) according to the manufacturer's instructions. GAPDH was used as an internal control. The primers are listed in Supplementary Table 2. For miRNA expression analysis, reverse transcription was performed using a ReverTra Ace qPCR RT Kit (Toyobo) with a miR-214 bulgeloop RT primer. The bulge-loop RT primer and qPCR primers specific for miR-214 were designed and synthesized by Guangzhou RiboBio (Guangzhou, China). The U6 small nuclear RNA was used as an internal control. Data analysis was performed using the $2^{-\Delta\Delta Ct}$ method.

According to standard Western blot procedures, briefly, proteins were separated by 8% SDS-PAGE and then transferred to nitrocellulose membrane (Bio-Rad). After blocking in 5% nonfat milk, the membranes were incubated with special primary antibodies. The proteins were visualized with Immobilon ECL (Millipore). The antibodies used include PLAGL2 (1:1000; Proteintech), MYH9 (1:1000; Proteintech), E-cadherin (1:1000; Proteintech), N-cadherin (1:1000; Proteintech), vimentin (1:1000; Proteintech), Zo1 (1:1000; Proteintech), P27 (1:1000; Cell Signaling Technology), cyclin D1 (1:1000; Cell Signaling Technology), cyclin E (1:1000; Cell Signaling Technology), CDK4 (1:1000; Proteintech), and GAPDH (1:1000; Proteintech).

Hematoxylin-eosin (HE) staining, immunohistochemical (IHC) staining and immunofluorescence (IF) staining

Complete sectioning was performed for all the human CRC tissues, orthotopic implantation tumors and metastases to ensure a precise diagnosis. 4µm-thick formalin-fixed and paraffin embedded sections were prepared for HE staining. Briefly, paraffin embedded sections were deparaffinized and rehydrated in a series of xylene and ethanol baths of decreasing concentration. Slides were put in hematoxylin solution for 1 min, followed by 1% alcoholic hydrochloric acid for 3 seconds, and then eosin solution for 1 min. Immunohistochemistry staining (IHC) was performed using a Dako Envision System (Dako, Carpinteria, CA) following the manufacturer's recommended protocol. For incubation with primary mAb, tissue slides were incubated at 4°C overnight. Negative controls were treated identically, but without the primary antibody. Immunofluorescence staining was performed on CRC cells and frozen sections of human CRC tissues. Cells were plated onto coverslips, washed with phosphate-buffered saline, fixed in 4% paraformaldehyde for 10 minutes, permeabilized with 0.25% Triton for 5 minutes and incubated with primary antibodies at 4°C overnight, followed by a 1-hour incubation with fluorescently conjugated secondary antibodies. Nuclei were counterstained with 4',6-diamidino-2-phenylindole (DAPI; CST), and then the coverslips were imaged via a confocal laser scanning microscope (FV1000; Olympus, Center Valley, PA).

Colony-formation assay

Twenty four hours after transfection, 500 uniparted cells were planted into 6-well plates and cultured for 2 weeks. Thereafter, the cell colonies were fixed with methanol for 5min and stained with 0.1% crystal violet for 15min at room temperature. Then cell colonies were counted and photographed.

EdU incorporation assay

For analysis of cell proliferation, 4×10^3 cells were seeded into 96-well plates. The cells were incubated under standard conditions in complete media. Forty-eight hours later, cell proliferation was detected using the incorporation of 5-ethynyl-2-deoxyuridine (EdU) with the EdU Cell Proliferation Assay Kit (RiboBio,

Guangzhou, China). Briefly, the cells were incubated with 50 μM EdU for 2 h before fixation, permeabilization and EdU staining, which were performed according to the manufacturer's protocol. The proportion of cells that incorporated EdU was determined by fluorescence microscopy.

Cell proliferation assay

The CCK-8 kit (Dojindo Laboratories Co. Ltd., Kumamoto, Japan) was used to assess cell viability in accordance with the manufacturer's introductions. In detail, we seeded cells (3×10^3 per well) into the 96-well plates with each well containing 200μl of culture medium supplemented with 10% FBS. We have six replicates for each sample. At the appointed time point, solution with 100μl of fresh medium and 10μl of CCK-8 solution was added into each well. After incubated for 1h at 37 °C, the absorbance was recorded at 450 nm using the Quant ELISA Reader (BioTek Instruments, USA). Survival rate % = (OD treatment−OD blank)/(OD control−OD blank) × 100%.

Migration assay

After transfection, cells were planted into 6-well plates and cultured for 24h. Then 200μl pipette tips were used to scratch three parallel lines and cells were washed with PBS twice, after which cells were cultured in an incubator at 37°C. Photographs were taken at 0 and 48h after wounding under Olympus FSX100 microscope (Olympus, Tokyo, Japan). Migration index was assessed by measuring the change of scratch area.

Invasion assay

Matrigel mix (BD Biosciences, CA, USA) was used to coat the top chamber of transwell chambers (8μm pore size) (BD Biosciences, CA, USA). Then 500μl DMEM containing 10% FBS was added to the bottom chamber while 1.5×10^5 cells were seeded in the top chamber. After 24h, cells that had passed through the matrigel to the underside of the filter were fixed with methanol and stained by 0.1% crystal violet, otherwise these were removed using cotton swabs. Cells were counted under Olympus FSX100 microscope (Olympus, Tokyo, Japan) and the number of stained cells represented invasiveness.

Scratch wound healing assays

We seeded the cells into the 6-well plates and cultured them until they fully fused. We then manually scratched the cell monolayer using a 200μl pipette tip and washed out the floating cells with phosphate-buffered saline (PBS). After that, cells were cultured for 48h in culture medium supplemented with 1% FBS. The phase contrast microscope (Niko Corporation) was used to capture the images and the Image Pro Plus v6.0 software package (Media Cybernetics Inc., Bethesda, MD, USA) was used to measure the migration areas of cells.

Dual-luciferase reporter assay

To verify whether miR-214 directly regulates PLAGL2 by combining the 3' UTR region of PLAGL2 mRNA, a dual-luciferase reporter assay (Promega, Madison, WI) was used. There are three potential binding sites in the 3' UTR region of PLAGL2 mRNA. Thus, wild-type and mutated 3' UTR sequence of PLAGL2 mRNA, called pGL3-PLAGL2-wild1–3 and pGL3-PLAGL2-mut1–3, respectively, were inserted between the hRluc and the hLuc genes in the pGL3 promoter vector. According to the manufacturers' protocols, HCT116 and SW480 cells were seeded in 96-well plates and then co-transfected with 100 ng of pGL3-PLAGL2-wild1–3/pGL3-PLAGL2-mut1–3 and 50 nM miR-214 inhibitor or the respective negative controls, using the RiboBio FECT™ CP Transfection Kit (RiboBio). The values of *Renilla*/firefly luciferase activities in the cell lysates were ultimately used for analysis.

Chromatin immunoprecipitation (ChIP)

ChIP assays were performed using the SimpleChIP® Plus Enzymatic Chromatin IP Kit (CST, USA) with anti-PLAGL2. Cells were crosslinked with formaldehyde and sonicated to an average size of 300-to-500 bp. Lysates were added to EP tubes, which were incubated with PLAGL2 antibody. Crosslinked DNA released from the protein-DNA complex was purified, and the eluted DNA was further detected by qRT-CR. Input and IgG were used simultaneously to confirm that the detected signals were derived from the specific bonding of chromatin and PLAGL2. All ChIP assays were repeated independently three times.

Animal experiments

For the xenograft subcutaneous implantation model, SW480 and HCT116 cells with different lentiviral transfection conditions were injected separately into five nude mice (8 weeks old). All mice were maintained for almost 30 days after injection. The sizes of the subcutaneous xenografts were recorded every 3 days.

For the pulmonary metastasis model, SW480 and HCT116 cells with different lentiviral transfection conditions were injected into the caudal vein of five nude mice (6 weeks old). All mice were maintained for almost 30 days after injection. Then, the pulmonary metastasis nodules were measured.
