# Supplementary material for: MicroRNA-214-3p targets the PLAGL2-MYH9 axis to suppress tumor proliferation and metastasis in human colorectal cancer
Source: Aging (Albany NY). 2020 May 15;12(10):9633–57. doi: 10.18632/aging.103233 (PMC7288958; doi:10.18632/aging.103233)
Supplement: Supplementary Figures [file aging-12-103233-s003..pdf]

SUPPLEMENTARY FIGURES

miRNA Name	Cancer Abbreviation	T-Test P-value	T-Test FDR	Upregulated in:	Tumor Log2 Mean Expression	Normal Log2 Mean Expression
hsa-miR-214-3p	BRCA	6.67e-05	1.78e-04	Normal	2.32	2.90
hsa-miR-214-3p	COAD	7.58e-04	2.79e-03	Normal	1.24	4.64
hsa-miR-214-3p	KICH	1.20e-10	2.73e-09	Normal	0.13	2.11
hsa-miR-214-3p	KIRC	8.05e-17	7.39e-16	Normal	0.59	2.19
hsa-miR-214-3p	KIRP	1.89e-17	1.24e-14	Normal	0.31	2.61
hsa-miR-214-3p	LIHC	2.86e-12	9.62e-11	Normal	1.09	3.05
hsa-miR-214-3p	PCPG	2.57e-02	3.52e-01	Normal	1.05	2.63
hsa-miR-214-3p	READ	4.40e-03	3.70e-02	Normal	2.21	5.41
hsa-miR-214-3p	STAD	1.06e-07	1.00e-06	Tumor	2.98	1.67
hsa-miR-214-3p	THCA	6.91e-17	3.36e-15	Normal	0.97	3.01

Supplementary Figure 1. The ONCOMIR data indicated that there are 10 cancer types in which tumorigenesis is significantly associated with the expression of miR-214-3p. The data are represented as the means±S.D. from at least three independent experiments. *$P<0.05$.

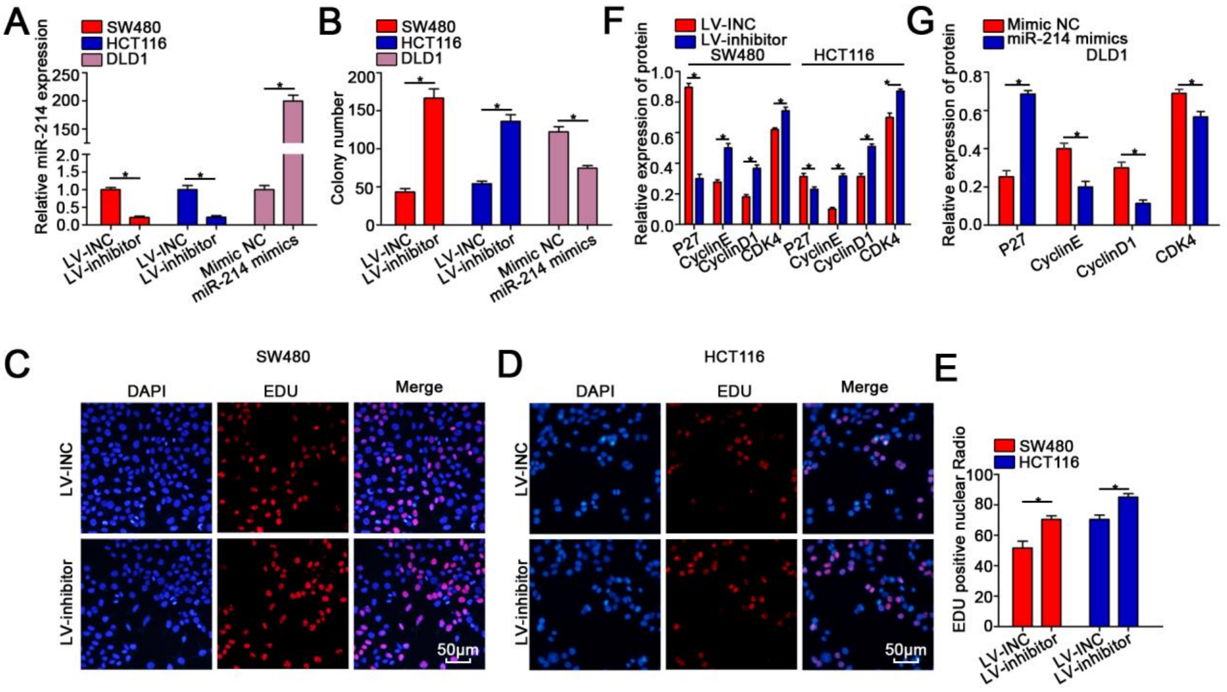

Supplementary Figure 2. (**A**) MiR-214-3p expression was significantly upregulated after transfection with the miR-214-3p mimic but downregulated after transfection with the LV-miR-214-3p inhibitor. (**B**–**E**) Colony-formation and EdU assays revealed that miR-214-3p suppresses CRC cell proliferation. (**F**–**G**) Western blot analysis revealed that miR-214-3p decreased the expression of cyclin D1, cyclin E and CDK4 and increased the expression of P27. The data are represented as the means±S.D. from at least three independent experiments. *$p<0.05$.

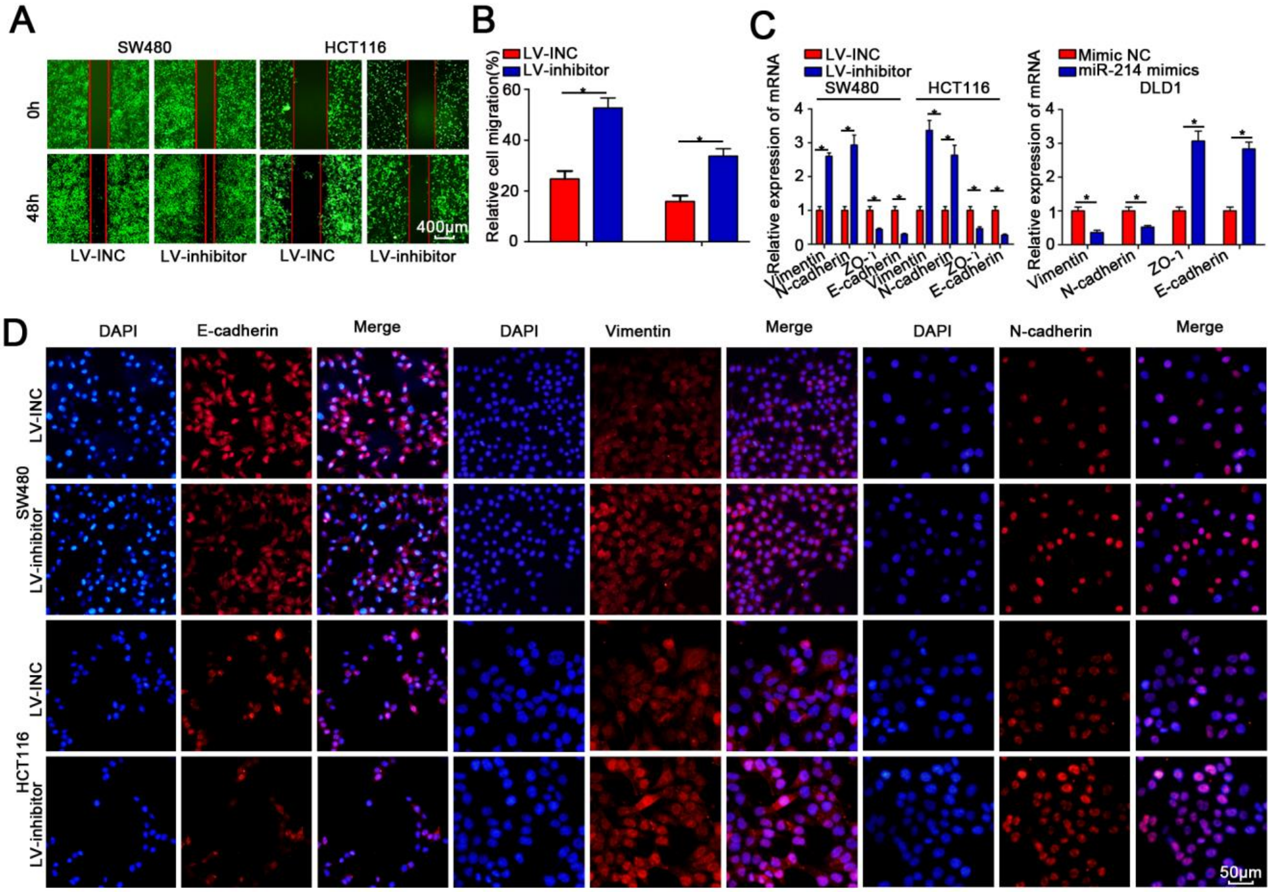

Supplementary Figure 3. (**A**–**B**) Wound-healing assays indicated that miR-214-3p suppressed the migration of CRC cells. (**C**) qRT-PCR assays showed that miR-214-3p decreased the expression of N-cadherin and vimentin and increased the expression of E-cadherin and Zo1. (**D**) IF assays showed that miR-214-3p decreased the expression of N-cadherin and increased the expression of E-cadherin. The data are represented as the means±S.D. from at least three independent experiments. *p<0.05.

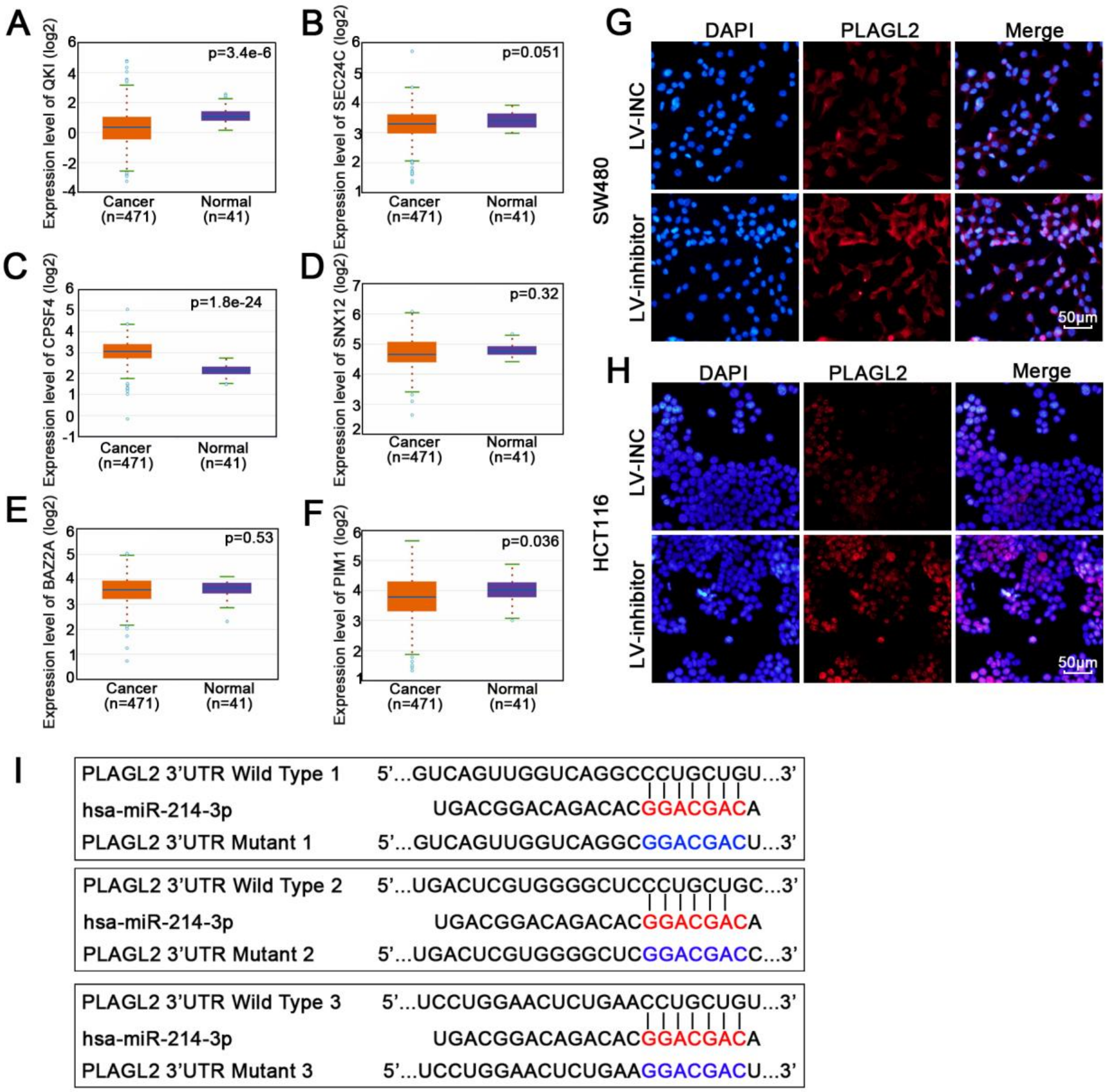

Supplementary Figure 4. (**A**–**F**) The expression levels of the potential targets of miR-214-3p according to the Starbase 3.0 database. (**G**–**H**) IF assays showed that miR-214-3p inhibited the expression of PLAGL2. (**I**) Predicted binding site in the 3'-UTR of PLAGL2. The data are represented as the means±S.D. from at least three independent experiments. **P*<0.05.

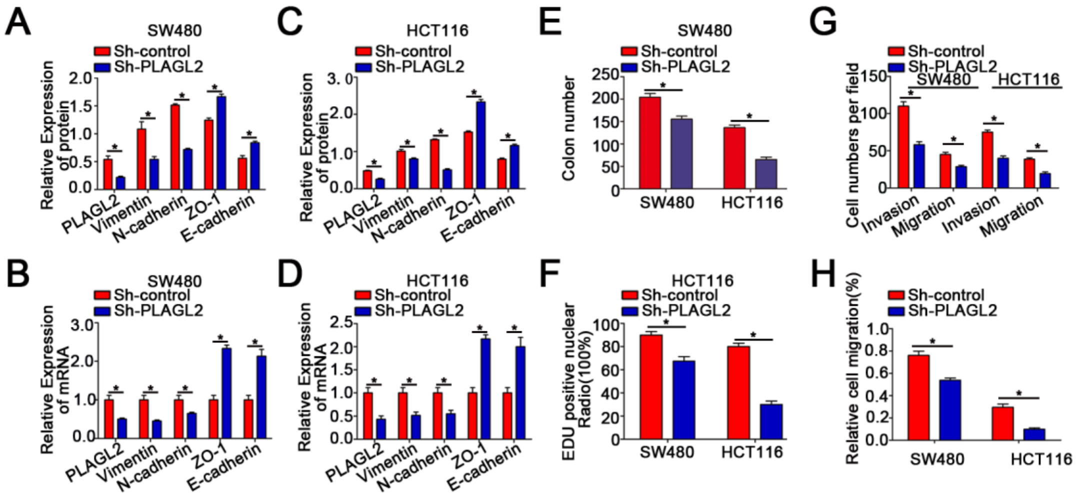

Supplementary Figure 5. (**A**–**D**) WB and qRT-PCR assays revealed that PLAGL2 increased the expression of N-cadherin and vimentin and decreased the expression of E-cadherin and Zo1. (**E**–**F**) EdU and colony formation assays revealed that PLAGL2 promoted CRC cell proliferation. (**G**–**H**) Transwell and wound-healing assays showed that PLAGL2 promoted the migration and invasion of CRC cells. The data are represented as the means±S.D. from at least three independent experiments. *P<0.05.

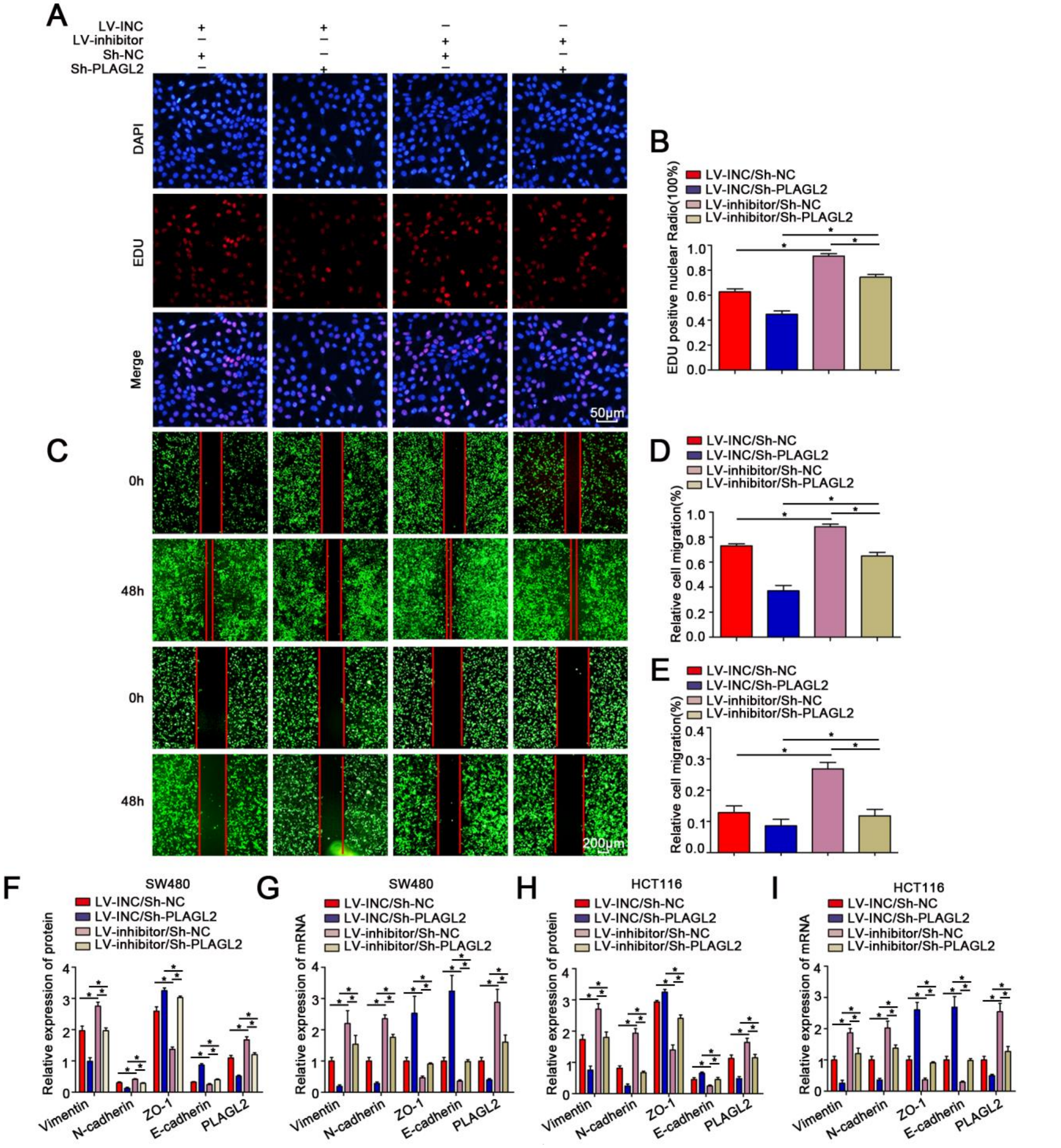

Supplementary Figure 6. (**A**–**B**) EdU assays revealed that PLAGL2 downregulation effectively reverses miR-214-3p inhibitor-induced CRC cell proliferation. (**C**–**E**) Wound-healing assays indicated that PLAGL2 downregulation effectively reverses miR-214-3p inhibitor-induced CRC cell migration. (**F**–**I**) Western blot and qRT-PCR assays indicated that the inhibitory effect of miR-214-3p on EMT was reversed by Sh-PLAGL2 transfection. The data are represented as the means±S.D. from at least three independent experiments. *$P<0.05$.

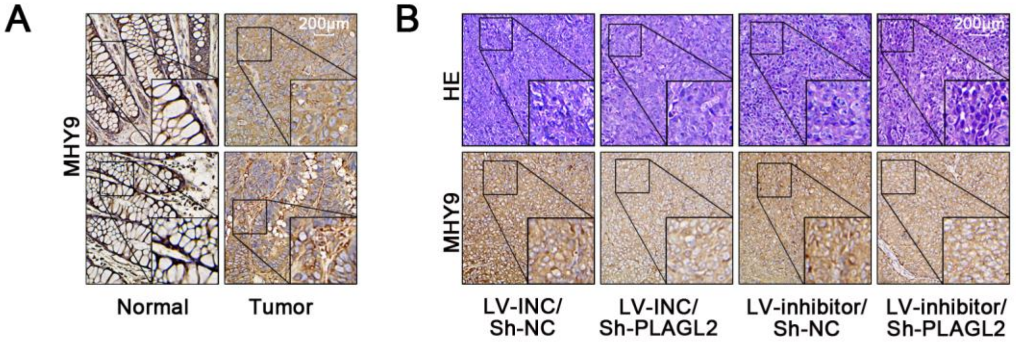

Supplementary Figure 7. (**A**) IHC analysis indicated that the expression of MYH9 was higher in tumor tissues. (**B**) IHC analysis indicated that the expression of MYH9 could be regulated by miR-214-3p and PLAGL2, and the inhibition effect of miR-214-3p on MYH9 could be reversed by Sh-PLAGL2 in subcutaneous xenograft tissues. The data are represented as the means±S.D. from at least three independent experiments. **P*<0.05.

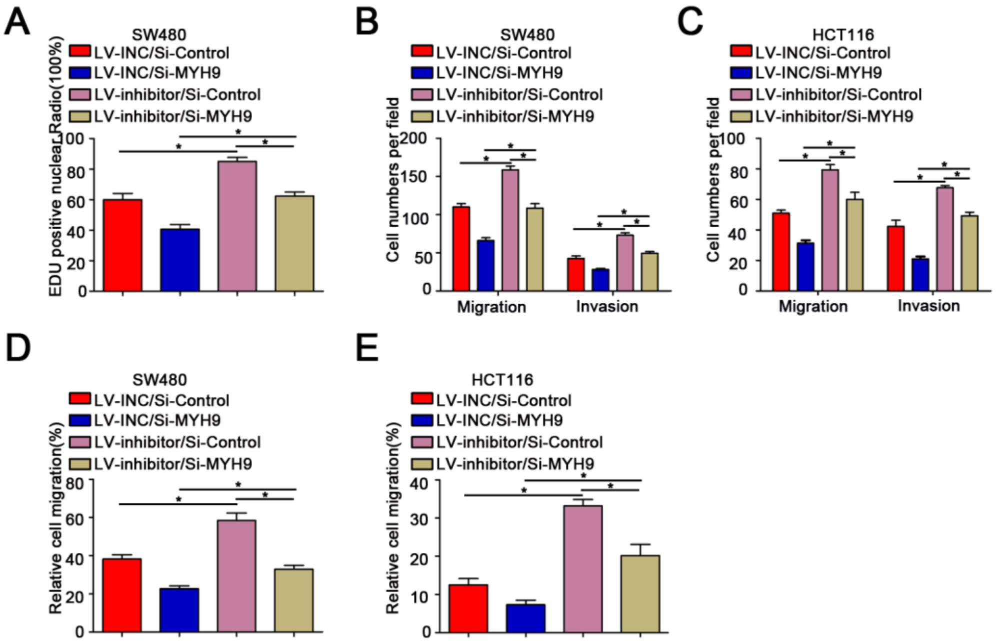

Supplementary Figure 8. (A) EdU assays revealed that MYH9 downregulation effectively reversed miR-214-3p inhibitor-induced CRC cell proliferation. (B–E) Transwell and wound-healing assays indicated that MYH9 downregulation effectively reversed miR-214-3p inhibitor-induced CRC cell migration. The data are represented as the means±S.D. from at least three independent experiments. **P*<0.05.
