# Supplementary material for: MicroRNA-214-3p targets the PLAGL2-MYH9 axis to suppress tumor proliferation and metastasis in human colorectal cancer
Source: Aging (Albany NY). 2020 May 15;12(10):9633–57. doi: 10.18632/aging.103233 (PMC7288958; doi:10.18632/aging.103233)
Supplement: Supplementary Tables [file aging-12-103233-s002..pdf]

SUPPLEMENTARY TABLES

Supplementary Table 1. Sequence information used in this study.

Gene	Sequence
miR-214 mimics	5'-ACAGCAGGCACAGACAGGCAGU-3'(sense)
	5'-ACUGCCUGUCUGUGCCUGCUGU-3'(antisense)
Mimic NC	5'-UUUGUACUACACAAAAGUACUG-3'(sense)
	5'-CAGUACUUUUGUGUAGUACAAA-3'(antisense)
miR-214 inhibitor	5′-ACUGCCUGUCUGUGCCUGCUGU-3'
Inhibitor NC	5'-CAGUACUUUUGUGUAGUACAAA-3'
PLAGL2 shRNA	5'-GACCCATGATCCTAACAAA-3'
MYH9 siRNA	5'-GACAGCAATCTGTACCGCATT-3'

Supplementary Table 2. Primers sequences.

Primer set	Primers	Sequence(5'-3')
**GAPDH**	Forward	GGGGAGCCAAAAGGGTCATCATCT
	Reverse	GACGCCTGCTTCACCACCTTCTTG
**N-cadherin**	Forward	CATCATCATCCTGCTTATCCTTGT
	Reverse	GGTCTTCTTCTCCTCCACCTTCT
**ZO-1**	Forward	CTGGTGAAATCCCGGAAAAATGA
	Reverse	TTGCTGCCAAACTATCTTGTGA
**E-cadherin**	Forward	GCCCTGCCAATCCCGATGAAA
	Reverse	GGGGTCAGTATCAGCCGCT
**Vimentin**	Forward	GCTTCAGAGAGAGGAAGCCGAAAA
	Reverse	CCGTGAGGTCAGGCTTGGAAA
**PLAGL2**	Forward	GAGGCCCTCAGCTCAGTTTT
	Reverse	GAGGCCCTCAGCTCAGTTTT
**MYH9**	Forward	ACCATGGAGGCCATGAGGATTA
	Reverse	CGATGTTGCCGAGCTGAAGA
**U6**	Forward	AAAGCAAATCATCGGACGACC
	Reverse	GTACAACACATTGTTTCCTCGGA
**miR-214**	Forward	GATGAGCTCAACTGAAGTGGCTAAAGAG
	Reverse	GATACGCGTTGAAGTTCTGCCTAATCTA
**1#**	Forward	AAAGGATGTATGCGAGTT
	Reverse	TTGTGAGCGTCTTGAGTT
